# Implementing a Community-Centered Approach to Gestational Diabetes Screening in Rural Guatemala: A Process Report

**DOI:** 10.3390/healthcare14030350

**Published:** 2026-01-30

**Authors:** Victoria Rabello Kras, Sasha Hernandez, Concepción Damián Chicajau, Josefa Damián Coquix, Rachel Siretskiy, Jessica Oliveira

**Affiliations:** 1 Saving Mothers, New York, NY 10022, USA; 2 Department of Obstetrics & Gynecology, New York University Langone Health, New York, NY 10016, USA; 3 Department of Obstetrics & Gynecology, University of Washington, Seattle, WA 98195, USA; 4 Herbert Wertheim College of Medicine, Florida International University, Miami, FL 33199, USA

**Keywords:** protocol implementation, low-middle-income countries, gestational diabetes, rural health, women’s health

## Abstract

**Introduction:** Gestational diabetes (GD) screening remains limited in many low- and middle-income countries (LMICs) due to resource constraints, limited training, and low community awareness. Although community-centered approaches may improve access to screening in rural and Indigenous settings, the implementation processes through which such approaches are designed and operationalized are rarely documented. **Methods:** This study presents a community-based implementation process report describing the development, adaptation, and early implementation of a GD screening program in rural Guatemala, guided by the Exploration, Preparation, Implementation, and Sustainment (EPIS) implementation science framework. Using a participatory approach, international screening guidelines were systematically adapted to the local context through iterative protocol refinement, structured stakeholder engagement, and ongoing feedback from community health educators and partner institutions. Aggregate program data were used descriptively to characterize early screening uptake and feasibility. **Results:** Key implementation challenges included patient no-shows, community skepticism, and difficulties among health educators in interpreting screening procedures. Iterative adaptations were introduced to simplify protocols, reduce loss to follow-up, and strengthen community engagement. Over time, the program expanded from point-of-care screening to more comprehensive prenatal services and increased collaboration with the Ministry of Health and local community outlets. A total of 103 Indigenous Mayan Tz’utujil women were screened (mean age: 26.9 years; range: 15–46), of whom, 12 were diagnosed with GD. **Conclusions:** This implementation process report demonstrates the scientific value of systematically documenting real-world adaptation, feasibility, and stakeholder engagement when introducing GD screening in rural Indigenous LMIC settings. The implementation lessons described may inform similar maternal health initiatives in comparable contexts.

## 1. Introduction

Gestational diabetes (GD), characterized by elevated blood sugar during pregnancy, poses severe risks to maternal, fetal, and neonatal health if not managed effectively [1,2]. Unregulated GD has been linked to the development of fetal congenital malformations, macrosomia, and respiratory distress while also significantly increasing the maternal risk of pre-eclampsia [1].

Despite international guidelines [3,4,5,6] on best practices for GD management in the intra and post-partum stages, GD remains a significant contributor to maternal and fetal morbidity and mortality. This is particularly concerning in low-middle-income countries (LMICs), where maternal and neonatal outcomes are worse when compared to high-income countries [1,2,7]. Furthermore, projections indicate a substantial increase in diabetes in LMICs by 2030, underscoring the critical importance of implementing effective management approaches to mitigate the health burden of this growing health challenge [1,8].

Adhering to current GD management guidelines and working toward improving GD screening and care in LMICs is accompanied by numerous challenges [9,10]. These hurdles include logistical complexities, such as the necessity of fasting visits to antenatal clinics, limited availability of quality lab infrastructure, and the elevated costs associated with these procedures [9,10]. In addition, there are cultural and social barriers such as the stigma attached to a GD diagnosis [9,10,11,12]. However, recent initiatives aimed at adapting international screening guidelines to address some of these challenges have demonstrated promising results in delivering care in LMICs [10,11,12]. They focus on adaptations that aim to overcome logistical barriers and resource limitations, potentially enhancing the effectiveness of GD screening and care in these settings.

Salvando Madres (SM) is a maternal health non-governmental organization (NGO) based in Lake Atitlán, Guatemala, led by local Mayan women. The organization is dedicated to enhancing awareness, access, and healthcare services for pregnant women. Their flagship program, the School of PowHer (Providing Outreach in Women’s Health and Educational Resources), trains traditional Mayan birth attendants (TBAs) to provide basic prenatal care and appropriate referrals for high-risk pregnancies. While their primary focus is on the School of PowHer, the SM leadership and School of PowHer students recognized the urgent need to address the increasing prevalence of diabetes they were encountering in their daily clinical practice. This aligns with the broader trend in Guatemala, a predominantly rural and indigenous LMIC, which has experienced a three-fold rise in diabetes cases [8].

With this in mind, SM is working to leverage financial, cultural, and social factors to implement initiatives aimed at increasing GD screening for local Mayan women. This implementation project report focuses on outlining our efforts to enhance GD screening by engaging stakeholders, adapting international guidelines to our context, and addressing implementation challenges. We detail the iterative process of adaptation and report on the challenges encountered during implementation in Lake Atitlán, Guatemala. Therefore, in this paper, we discuss solutions and strategies for addressing barriers and challenges associated with implementing effective GD screening protocols in LMICs with the hopes of providing an effective framework for the development of such initiatives in other LMIC settings.

### Rationale

In 2021, Guatemala’s maternal mortality rate (MMR) reached 121 per 100,000 live births, driven largely by hemorrhage and hypertensive disorders, which disproportionately affect Mayan women compared with Ladina women [13]. Infant mortality was 19.9 per 1000 live births in 2019, and the stillbirth rate was 13.3 per 1000 in 2021 [13]. Despite ongoing national efforts, the country remains behind in achieving the Sustainable Development Goal of reducing maternal mortality to fewer than 70 per 100,000 live births [14]. These persistent disparities underscore the need to address pregnancy-related conditions, such as GD, that contribute to maternal complications and elevated MMRs [15,16].

## 2. Materials and Methods

This manuscript presents an implementation process report describing the development, contextual adaptation, and early introduction of a community-centered GD screening protocol in Santiago Atitlán, Guatemala. The study is guided by the Exploration, Preparation, Implementation, and Sustainment (EPIS) implementation science framework [17] and focuses on systematically documenting implementation processes, contextual determinants, and stakeholder engagement that shaped protocol adaptation and application in a rural Indigenous setting.

The implementation project emerged from programmatic observations within a community-based maternal health program serving Indigenous women in Santiago Atitlán. As the program encountered increasing numbers of pregnant women with clinical risk factors for diabetes mellitus, attempts to facilitate referral for GD screening revealed substantial system-level gaps. At the time of project initiation, no standardized GD screening or diagnostic protocol existed across local health facilities. Screening practices, diagnostic thresholds, testing capacity, and referral pathways varied by facility and provider, and no consistent system for follow-up or management of GD was in place. These contextual conditions defined the implementation problem addressed by this project.

**Figure 1:**
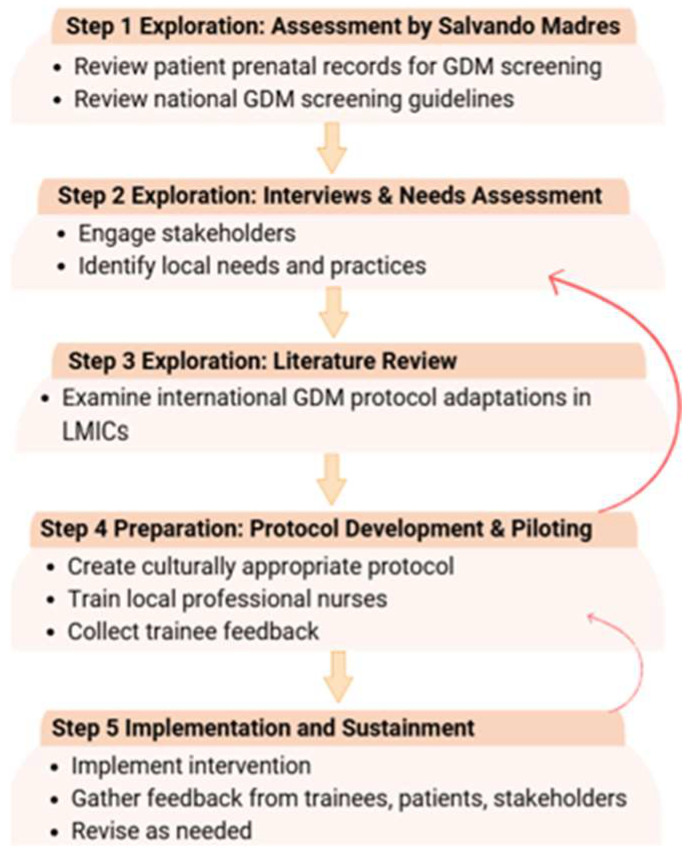
Intervention mapping process. This figure presents the EPIS-guided implementation process in a vertical, stepwise format. Curved arrows indicate iterative feedback loops between phases, reflecting the adaptive nature of implementation in real-world settings.

In response, a local clinical and community health team initiated the systematic development, adaptation, and documentation of a context-appropriate GD screening protocol, with the explicit aim of generating implementation knowledge relevant to similar low-resource settings with an elevated risk for type 2 diabetes. The EPIS framework was selected to structure this work due to its emphasis on multilevel context, stakeholder engagement, iterative adaptation, and real-world feasibility. These features make the EPIS framework particularly well suited for implementation research in LMICs, where health system constraints and variability necessitate flexible, partnership-driven approaches. The application of the EPIS framework across the different project phases is summarized in Figure 1.

### 2.1. Pre-Implementation: Step 1 (Exploration)

**Figure 2:**
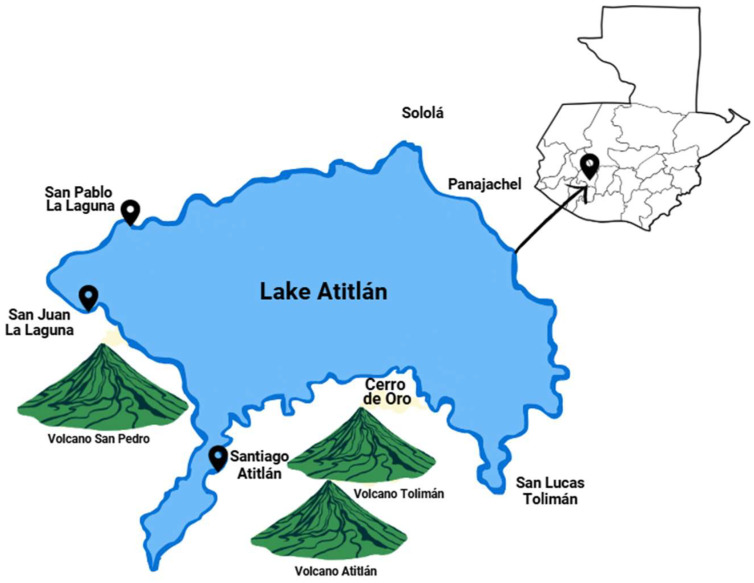
Study setting and communities assessed during the pre-implementation (exploration) phase. Map of the Lake Atitlán region showing communities included in the pre-implementation assessment of gestational diabetes screening practices. Geographic mapping informed the identification of contextual and health system factors influencing screening and referral.

The pre-implementation phase lasted six months during which time our local team was able to (1) map the available GD screening services and (2) the current GD screening guidelines being used. We identified a total of six health centers offering GD screening. See Table 1 for a summary of the screening practices at each health center. Figure 2 presents a map of Lake Atitlán, indicating the geographical locations of the towns included in the assessment.

The official Guatemalan Ministry of Health and Public Health and Social Assistance (MSPAS) guidelines recommend three glucose screenings during pregnancy (<24 weeks gestation, 24–28 weeks, and 32–34 weeks) to detect the presence of diabetes mellitus in pregnancy or GD [18]. As per the guidelines, any point-of-care (POC) blood glucose result ≥92 mg/dL at any testing date should be referred to the hospital for confirmation with an oral glucose tolerance test (OGTT) and further classification [18].

Although all identified health centers are making great efforts to address GD, they each employ different and inconsistent screening protocols that do not adhere to the recommended MSPAS guidelines. Most of the health centers reported a lack of staff, lack of staff training, and lack of resources as limitations in testing for and identifying GD. When and if identified, GD cases are referred to the National Hospital of Sololá (NHS) in Sololá for management; however, this service was unavailable due to the COVID-19 pandemic and has not yet been fully reinstituted. The NHS, despite being free of charge, faces challenges such as high patient volumes, limited staff, long waiting times, and difficult access due to geography.

### 2.2. Development of Original Protocol: Steps 2–4 (Preparation)

Following an extensive literature review, several studies were identified that successfully adapted international GD screening guidelines for use in LMICs using POC fingerstick glucometer tests, demonstrating positive outcomes despite resource limitations and logistical challenges [10,11,12]. Although not the standard of care for high income countries, WHO and FIGO have also advocated for the use of glucometers for screening and diagnosing GD in LMICs [3,4]. This information was coupled with an assessment of GD screening guidelines and adherence in multiple clinics around Lake Atitlán, as previously described. A screening protocol was therefore designed and adapted to be sustainable and effective within the context of Santiago Atitlán, which included balancing challenges such as limited resources, inadequate infrastructure, and adherence issues.

**Figure 3:**
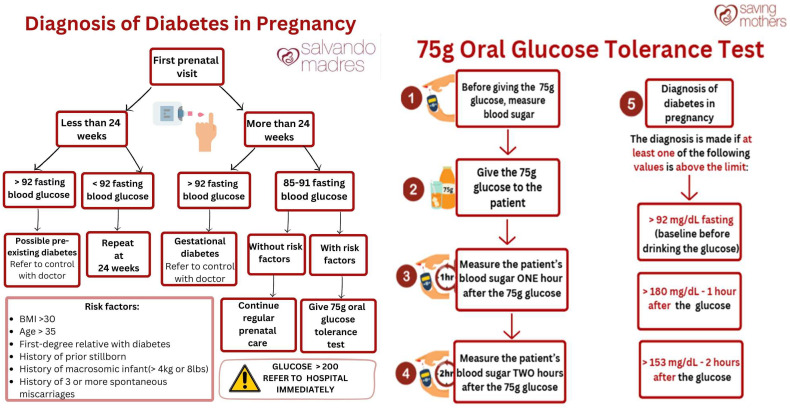
Original protocol.

Therefore, we elected to use glucose measurements obtained from capillary whole blood using a glucometer that reports plasma-equivalent values (ACCU-CHEK, Indianapolis, IN, USA; glucometer and test strip make and model). Known risk factors for GD, including a first-degree relative with any type of diabetes mellitus, BMI > 30 kg/m^2^, age > 35 years, history of GD, history of ≥3 miscarriages, previous stillbirth, and macrosomia are tentatively documented for every woman screened. Weight and height are also measured and documented. A diagnosis is made if the fasting blood glucose (FBG) is ≥92 mg/dL. Women receiving their first screening at <24 weeks with FBG ≥ 92 mg/dL are considered to have preexisting diabetes and are referred to an MOH clinic for management. Those screened at <24 weeks with an FBG < 92 mg/dL are scheduled for repeat POC testing after 24 weeks. For women screened at >24 weeks, an FBG ≥ 92 mg/dL results in a diagnosis of GD and referral to an MOH provider. If the FBG is 85–91 mg/dL and no risk factors are present, routine prenatal care continues. If a ≥1 risk factor is present, an oral glucose tolerance test (OGTT) is required. For the OGTT, women present to the office the next day in a fasting state. Fasting POC glucose is measured, a 75 g glucose load is administered, and glucose is measured again at 1 h and 2 h. GD is diagnosed if the FBG ≥ 92 mg/dL, 1 h glucose ≥ 180 mg/dL, or 2 h glucose ≥ 153 mg/dL. Fasting status was ensured by instructing women to attend appointments in a fasting state prior to the appointment, which was confirming verbally upon their arrival. See Figure 3 for the original protocol.

If diagnosed with GD, the expectant mother receives comprehensive education on various aspects of the condition, including its pathogenesis, risk factors, diagnostic criteria, and potential complications for both mother and baby. Women are provided with detailed guidance on maintaining a healthy diet and exercise regimen. For further management, they are referred to the MOH for specialized care and support with a provider and nutritionist. When the original protocol was first created and implemented, local leadership of SM engaged with the director of the local MOH clinic to present the protocol and coordinate screening schedules. This meeting sought to establish a partnership and foster ongoing collaboration between SM and the MOH. It also aimed to inform the director and equip him to relay these details to his team, ensuring that pregnant women in the community were aware of the free glucose screenings available to all expectant mothers. The MOH was supportive and welcoming of the initiative, expressing willingness to collaborate with SM in introducing this innovative GD screening program to the broader community.

### 2.3. Initial Staff Training: Step 4 Preparation

Two SM staff members were designated to receive training on GD and the protocol to lead the GD project. Both are nurses and had previously participated in the first PowHer School cohort. The training included PowerPoint presentations covering the basic pathophysiology of GD, associated risk factors, diagnostic criteria, maternal and neonatal complications of untreated GD, and recommended management. Although both nurses were already familiar with glucometer operation, they received additional instruction on interpreting glucometer readings according to the protocol, and proper handling of the device. They were also trained to apply the protocol decision pathways, including determining the appropriate next steps based on the woman’s gestational age and her FBG value.

Quality control procedures included ensuring that all staff were trained in proper fingerstick technique, performed hand hygiene and glove use prior to sample collection, and adhered to the standardized protocol, including only sampling women in a confirmed fasting state. When erroneous or inconsistent readings were observed, troubleshooting involved restarting the glucometer and repeating the measurement with a new test strip. Additionally, bimonthly meetings with SM leadership were conducted to review screening processes and address any identified gaps in testing procedures or protocol interpretation. Finally, six months into protocol implementation, a designated point person was appointed to support protocol fidelity by overseeing implementation, providing on-site supervision, assisting with troubleshooting during GD clinics, and helping adapt the protocol to enhance its sustainability.

### 2.4. Ethics Statement

The research was reviewed by New York University Langone Health Institutional Review Board and received an exemption under 45 CFR 46.104 (d) for secondary analysis of de-identified program records.

## 3. Results

### 3.1. Testing: Step 5 Implementation

All quantitative results presented within this manuscript represent de-identified aggregated program data. As these data do not originate from a research cohort and are not intended to support inferential conclusions, the analysis was limited to descriptive counts only, without confidence intervals or other inferential measures.

**Figure 4:**
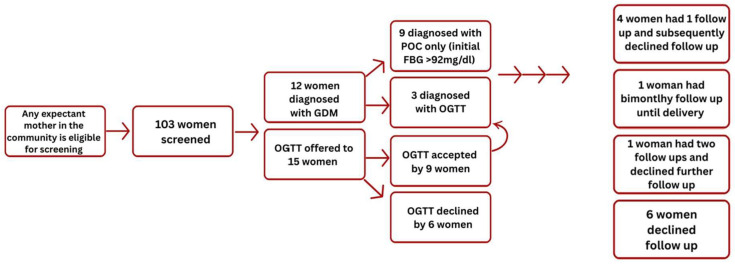
GD testing flow diagram. GDM = gestational diabetes mellitus; POC = point-of-care; OGTT = oral glucose tolerance test; FBG = fasting blood glucose.

Since March 2023, GD testing has been conducted at the SM office in Santiago, Atitlán. Testing is free for all expectant mothers who had fasted overnight. Accordingly, all pregnant women in the community, including those receiving prenatal care from SM TBAs or affiliated elder TBAs, those referred by the local MOH clinic, and those who independently learn about the initiative and presented for services, are eligible to undergo GD screening. From March to August 2023, POC screenings were conducted on 32 women, 2 were identified to have GD and were lost to follow up, and no OGTT were administered during this period. In September 2023, a senior medical student joined the SM team to advance the GD initiative, and from September 2023 to August 2024, 71 women were screened, 12 cases of GD were identified (9 meeting the criteria for diagnosis with POC screening only and 3 meeting the criteria with OGTs), 9 OGTTs were administered, and a total of 103 women were screened. Of the 12 women diagnosed with GD, 6 declined further follow up, and information regarding pregnancy outcomes, labor characteristics, and neonatal status was not available. Among the remaining six women, four delivered macrosomic infants (birth weight > 8 lbs), one woman developed hypertensive disorder of pregnancy, and two required cesarean delivery (both involving macrosomic infants). One cesarean was performed for failure to progress in labor, while the other was an elective procedure. See Table 2 for a summary of our de-identified aggregated GD screening program data. Refer to Figure 4 for the program flow diagram.

### 3.2. Challenges

#### 3.2.1. Loss to Follow-Up

Although 25 women completed both screenings, 78 only underwent a single screening (<24 weeks or ≥24 weeks GA), with 13 undergoing the <24 weeks screening and explicitly refusing the follow-up test at ≥24 weeks. For women diagnosed with GD, weekly monitoring during the first one to two weeks was largely successful; however, most subsequently declined further follow up. Efforts for re-engagement were conducted via phone calls and home visits by TBAs with variable success depending on phone access, signal quality, and women’s ability to attend scheduled appointments.

The leadership at SM hypothesizes that the inadequate follow-up during both the screening phase and subsequent monitoring of GD is multifactorial. Efforts to reach women by phone were hindered by many lacking mobile devices or poor signal quality when devices were available. However, in most cases, successful contact was established, either by phone or through TBAs, and many women verbally committed to attending their next appointment yet failed to follow through, whereas many women declined further follow up altogether. This behavior reflects a broader cultural tendency within the community, where individuals may avoid direct refusal and instead prioritize expressions of gratitude and politeness, even if there is no intention to fulfill the commitment. Other significant factors contributing to loss of follow-up, as stated by many declining follow-ups, are geographical limitations, lack of reliable transportation, and time. This poses a significant barrier for women living far from our clinic or in neighboring towns as they are required to travel to the office for testing and pay for transportation both ways. It is worthwhile mentioning that most of our target population come from economically disadvantaged backgrounds and are solely responsible for domestic duties and caring for other children in the home. Consequently, transportation costs and especially the time required away from household and childcare responsibilities were significant and prevalent barriers that impacted women’s adherence to screenings.

Cultural interpretations and limited acceptability of the testing procedures also influenced some women’s willingness to return for follow-up. Many expressed skepticisms about the screening, noting that these tests were not performed at the MOH clinic, where they receive most of their prenatal care alongside care provided by our affiliated TBAs. POC fingerstick glucose testing and the OGTT were unfamiliar to many women, as such procedures are not typically included in standard prenatal care in rural regions of Guatemala. This unfamiliarity contributed to uncertainty about the screening program. Additionally, when advised to undergo the OGTT, many women reported difficulty committing to the required two-hour waiting period due to pressing household responsibilities.

Furthermore, within this community, many women depend on their husbands for healthcare decision-making, and several reported receiving inadequate support from their husbands or other family members, contributing to their decision to decline further follow-up.

#### 3.2.2. Lack of Community Knowledge of GD

For the community of Santiago Atitlán, where GD screening is not standard practice for prenatal care, a significant knowledge gap around GD exists, both at the provider and patient levels. This is compounded by insufficient government efforts to promote awareness of GD and limited education provided to women during prenatal consultations. Although the average Guatemalan woman has 2.3 children throughout her reproductive years, and most women reached through our initiative are multigravida, many are introduced to GD for the first time during their screenings at the SM [19]. Additionally, this is a community with strong cultural health beliefs in which alternative explanations and management strategies for physiological events are commonly followed, which is observed in many rural and indigenous towns in LMICs [20,21]. For example, some women perceive elevated blood glucose levels as resulting from fear or other external factors and manage these with natural remedies. Taken together, these structural, educational, and cultural factors ultimately diminish women’s likelihood of accepting GD screening or engaging in recommended follow-up.

#### 3.2.3. Distrust in Medical Providers

Many women also express worry and skepticism about seeking medical care or undergoing tests recommended by physicians and nurses. Much of this hesitation stems from a broader distrust of the medical system, shaped by negative personal experiences as well as those of family and friends. Women frequently report feeling that clinic and hospital providers do not care for them adequately and often come across as dismissive. These perceptions are further reinforced by the association of healthcare facilities with illness and death, which heightens patient apprehension. Historically, this community has favored TBAs over clinical settings for prenatal care and delivery [22,23]. Within the Mayan community, TBAs are believed to be chosen by God through visions or dreams and to receive their abilities through divine means [24]. As a result, they hold a level of respect and trust that surpasses that afforded to medically trained professionals. Correspondingly, during protocol implementation, adherence to screening and follow-up improved markedly when TBAs were engaged, through GD education and scheduling support, compared to when encouragement was delivered solely by the SM nursing staff.

#### 3.2.4. Difficulty Counseling

Despite initial staff training prior to protocol implementation, the SM nursing team demonstrated limited understanding of the rationale and importance of GD screening at the onset of the rollout. This gap contributed to inadequate communication of key GD concepts to the women screened. Uncertainty regarding the appropriate glucose ranges and diagnostic cutoff values resulted in misinterpretations and missed opportunities to administer the OGTT during the early months of implementation. This lack of clarity also affected staff confidence in counseling women and explaining next steps. Collectively, these issues contributed to delays in follow-up and diagnosis during the first six months of protocol implementation.

#### 3.2.5. Additional Challenges

Outreach efforts are also limited by the high cost of glucometers and associated supplies, allowing for only one glucometer at the SM centralized office. This requires women to travel to the SM office for screening, as we were unable to offer it during home prenatal visits. Further, SM TBAs performing home prenatal visits would need specialized training if they were to conduct GD screenings during their home prenatal visits. This training presents logistical and resource challenges, including the time and expertise required to ensure accurate and consistent screenings. Lastly, the protocol requires women to fast prior to screening, which is impractical during home visits. TBAs travel extensively between towns and homes daily, making it difficult to ensure that women remain fasting for the necessary duration.

Furthermore, when a woman is diagnosed with GD by the local MOH clinic, they are referred to the NHS for further management. This referral system, however, faces significant challenges. Traveling to Sololá from surrounding communities is geographically difficult and time-consuming, limiting patients from following through with medical care. As the main public hospital serving the entire department of Sololá, the NHS frequently struggles with resource limitations, prolonged waiting times due to staff shortages, and high patient volume, all of which are common complaints we heard from women.

### 3.3. Adaptations for Sustainment (Step 5)

#### 3.3.1. Education

Suboptimal interpretation and comprehension of the protocol by the local staff responsible for conducting GD screening led to inconsistent implementation and adherence during the initial months of protocol rollout. To address this challenge, a GD project coordinator, who was also a senior medical student, was appointed as the point person. The coordinator directly participated in screening activities to ensure accurate protocol interpretation and conducted monthly protocol reviews. These reviews incorporated nurse feedback and resulted in protocol modifications aimed at improving clarity, enhancing staff understanding, and tailoring it to the needs and practical realities of the target community. Additional training was also delivered through PowerPoint presentations to the six SM TBAs who conduct prenatal and postpartum home visits. This training emphasized core GD education, addressed common misconceptions, and equipped TBAs with educational flyers on GD and healthy lifestyle practices to support counseling and recruit women forscreening . Recognizing the influence of elder TBAs in rural communities such as Santiago Atitlán, targeted educational sessions were also provided to them, with the expectation that their involvement would enhance community-level dissemination of GD information and promote increased screening participation.

Low community awareness of GD and limited uptake of the initiative underscored the critical need to prioritize community education. This approach is supported by the literature, which indicates that educational interventions are both effective and cost-efficient strategies for addressing non-communicable diseases in LMICs [25]. In response, SM partnered with a local health television program, where staff participated and presented information on GD, and also aired short informational segments and lifestyle guidance on a local radio station. To ensure broad accessibility and comprehension, all content was delivered in both Spanish and the local Mayan dialect. During both the television programs and radio segments, announcements were also made to inform the community about the free GD screening available at the SM office. Additionally, SM partnered with the local MOH clinic to deliver group educational talks to pregnant women during ultrasound days, further promoting community awareness of GD and reinforcing lifestyle modifications, such as increasing physical activity and reducing carbohydrate intake, to support healthier pregnancies.

#### 3.3.2. Adaptations to the Original Protocol

**Figure 5:**
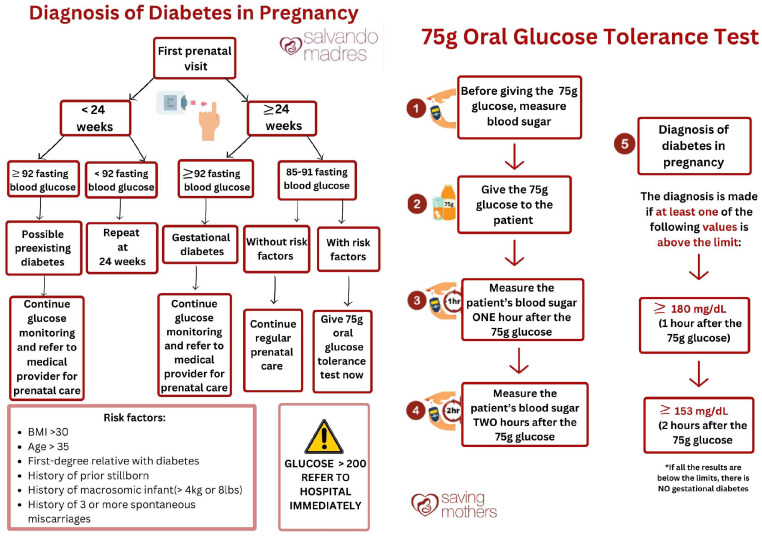
Current working protocol.

Several modifications to the original protocol were required to address the challenges encountered. Initially, the protocol required that women at >24 weeks with a fasting blood glucose level between 85 and 91 mg/dL and the presence of at least one risk factor return the next day for a repeat POC fingerstick test and possibly an OGTT. A diagnosis of GD would be made if any of the following cut-off values were exceeded: >92 mg/dL fasting, >180 mg/dL 1 h post-OGTT, and >153 mg/dL 2 h post-OGTT. However, a high proportion of women did not return for the second confirmatory test, stating they were unable to take extra time off their household and childcare duties. To address this and improve follow-up compliance, the protocol was modified to administer the OGTT immediately to women with a fasting blood glucose range of 85–91 mg/dL and at least one risk factor, eliminating the need for a return visit. This modification significantly reduced loss to follow-up and ensured that all women requiring an OGTT received it for confirmation or to rule out GD. See Figure 5 for protocol adaptations.

Another significant protocol adjustment involved the referral process. Initially, all women diagnosed with GD were referred to the MOH clinic for follow-up, monitoring, and management. However, limited engagement from MOH leadership and suboptimal dissemination at the clinic contributed to low protocol uptake, leading to women receiving mixed messages from SM and the MOH clinic regarding their GD status. To ensure identified women with GD are monitored, SM now offers weekly or bi-monthly finger stick tests for ongoing blood glucose monitoring depending on individual availability and proximity to the office. This monitoring is complemented by blood pressure and weight checks for continued assessment. Additionally, these visits provide education on maintaining a healthy diet and weight during pregnancy, focusing on increasing physical activity and decreasing carbohydrates. While glucose monitoring for those diagnosed is now offered through POC checks at the office, women are also provided with a referral slip, outlining their diagnosis based on the working protocol, to present to the doctor at the MOH clinic and ensure continued communication and collaboration between SM and the MOH in the care of these women.

#### 3.3.3. Testing Adaptations

Given that community reach in Santiago Atitlán was lower than initially anticipated, we sought to enhance GD screening coverage, improve detection rates, and increase regional awareness of GD. Accordingly, monthly glucose testing services were introduced in October 2023 in Cerro de Oro, a neighboring town identified for its notably high pregnancy and birth rates within the region. During each visit, 2–6 women were successfully screened, increasing the overall population SM reaches. Additionally, we have formed a partnership with an MOH clinic in the neighboring town of San Lucas Tolimán, with the hopes to initiate monthly screening there in the future.

In July 2024, to further incentivize participation in GD screening, appointments were expanded into a comprehensive prenatal care clinic at our central facility. This shift was driven by the recognition that women highly value hearing their baby’s heartbeat, viewing ultrasounds, and receiving reassurance about their pregnancy. As a result, GD screening days now include not only glucose screening but also routine prenatal assessments such as vital sign monitoring, anthropometric measurements, and fetal evaluations, including fetal heart rate assessment using fetal Doppler, fundal height measurement, and fetal position assessment. These visits also serve as opportunities to educate women on GD, healthy eating habits, lifestyle modifications, warning signs of pregnancy requiring medical attention, and the importance and distribution of prenatal vitamins. Finally, the two staff members responsible for the GD initiative are currently undergoing training in obstetric ultrasound, which will soon be incorporated into these visits. Because women frequently request ultrasound examinations, integrating this service is expected to further encourage attendance and, in turn, increase uptake of POC glucose screening and monitoring when offered during the same appointment.

## 4. Discussion

This implementation process report examines the development and early introduction of a context-adapted GD screening protocol in a rural Indigenous community in Guatemala using an iterative, community-centered approach. The primary objective of this discussion is to interpret implementation challenges and adaptations encountered during protocol rollout and to identify implications for sustainment and replication in similar low-resource settings, rather than to evaluate clinical effectiveness.

Consistent with prior literature, our findings underscore the importance of adapting established screening protocols to the local health system capacity, sociocultural norms, and community trust structures rather than applying international guidelines without modification [26]. Although the initial protocol was designed to address known resource constraints, ongoing refinement was required throughout implementation. This iterative adaptation process, central to the EPIS framework, enabled the program to respond to emerging barriers, refine workflows and messaging, and improve feasibility over time.

Several interrelated challenges shaped implementation. Loss to follow-up, limited community awareness of GD, distrust of the formal healthcare system, and early difficulties among staff responsible for counseling and protocol interpretation, all constrained screening uptake and continuity of care. As described in the Results, follow-up was limited both for completion of recommended repeat screening and for monitoring after diagnosis. Barriers such as transportation costs, geographic distance, time away from domestic responsibilities, and childcare demands have been widely reported in LMIC settings and were prominent in this context [7,9,27,28,29]. These challenges were further exacerbated by the novelty of fasting-based screening, and the time demands of OGTT, which have been shown to limit adherence in resource-constrained settings [9,28,29]. Protocol adaptations aimed at reducing repeat visits and expanding access points were therefore essential to maintaining feasibility.

Low uptake also reflected substantial gaps in health literacy related to GD. General health literacy remains limited in many Latin American countries and is particularly low in Guatemala, disproportionately affecting rural and Indigenous populations [23,30]. Limited understanding of GD contributed to fear, stigma, and anxiety surrounding screening and diagnosis. Inconsistent public messaging, constrained MOH outreach capacity, and the absence of standardized national implementation of GD screening reinforced perceptions that GD is not a priority condition [7,31]. To address this, educational outreach was strengthened through locally trusted channels, including prenatal counseling, collaboration with local media, and coordination with MOH-led community talks.

Distrust of the formal healthcare system emerged as a critical determinant of screening acceptability and follow-up. Women frequently cited negative personal and shared experiences with facility-based care, including perceived dismissive treatment, poor-quality services, and fear of medical interventions such as cesarean delivery [7,23,31]. In contrast, TBAs hold longstanding cultural legitimacy within Mayan communities and are often preferred sources of prenatal care [22,23,24]. This trust dynamic directly influenced implementation: adherence to screening and follow-up improved when TBAs were actively engaged in education and scheduling support compared with outreach led solely by nursing staff. These observations align with prior evidence demonstrating the importance of trusted community health workers in maternal health interventions in similar settings [23,32].

Finally, early challenges among nursing staff in interpreting screening protocols highlighted that implementation success requires more than written guidelines or diagnostic tools. Initial uncertainty regarding diagnostic thresholds and referral pathways limited effective counseling and follow-up. The introduction of structured supervision, including designation of a clinical point person to support protocol interpretation and iterative refinement improved consistency and staff confidence. Together, the combination of trusted community engagement and structured supervisory support was central to strengthening protocol fidelity and program sustainability.

Comparable implementation challenges have been documented in other low-resource and rural contexts, including rural Turkey and the Peruvian Amazon, where logistical constraints, patient reluctance, and the need for locally tailored strategies similarly influenced GD screening efforts [33,34,35]. Although published descriptions of GD screening implementation in Latin America remain limited, experiences from maternal health initiatives in rural Guatemala further reinforce the importance of community engagement, iterative adaptation, and service integration when introducing new screening programs [35].

### 4.1. Directions for Continued Sustainment

While community education remains essential to improving GD screening uptake, long-term sustainment of this initiative also depends on strengthening engagement and capacity among healthcare providers, particularly auxiliary nurses and MOH staff involved in prenatal care. During implementation, inconsistent understandings of GD and its maternal and neonatal implications, along with limited familiarity with screening guidelines, contributed to mixed messaging and variable provider buy-in. Although community-based protocols can mitigate access barriers, sustainable integration requires alignment between community outreach and facility-based care. Strengthening provider education within the constraints of local resources and referral pathways is therefore critical to ensuring coherent counseling, reinforcing screening recommendations, and supporting long-term program sustainability.

### 4.2. Future Perspectives

Scaling this initiative may inform broader strategies within Guatemala’s national healthcare system by aligning community-centered screening models with national maternal health policies. Integrating GD screening into designated prenatal care days at MOH clinics, alongside continued testing at the SM office, could reduce transportation and time burdens while minimizing conflicting diagnoses. This approach would allow women to receive routine prenatal care and glucose screening during a single visit, though coordination with MOH leadership would be necessary to address long wait times and preserve fasting conditions.

Leveraging family and community dynamics also represents an opportunity to improve screening adherence. In this setting, male family members often play a central role in healthcare decision-making, and limited spousal support emerged as a barrier to follow-up. Targeted education for male partners regarding GD and its maternal and neonatal implications may strengthen support for prenatal care and screening participation. Evidence from other LMICs suggests that male involvement in maternal health education is associated with improved prenatal care utilization and pregnancy outcomes [29,36,37,38].

Dietary modification remains central to GD prevention and management, but counseling must be culturally appropriate and economically feasible. In communities where maize-based foods are dietary staples, nutritional guidance requires sensitivity to cultural norms and household constraints [39,40]. While emerging preventive strategies such as probiotic supplementation have shown modest metabolic benefits in higher-resource settings [41], their feasibility in this context is limited. Locally acceptable alternatives, including traditional fermented foods, may warrant future exploration. Together, these perspectives highlight the importance of context-driven, multisectoral approaches to sustaining and expanding GD screening initiatives in rural Indigenous settings.

### 4.3. Perspectives for Clinical and Practice Implications

From a clinical and practice perspective, this implementation process report highlights the importance of aligning screening protocols with real-world health system capacity and community trust structures. In low-resource rural settings, effective GD screening requires simplified workflows, clear counseling tools, and integration with existing prenatal care services rather than reliance on complex diagnostic pathways. Engagement of trusted community health workers, including TBAs, can enhance acceptability, support informed decision-making, and improve continuity of care.

For practitioners and program implementers, these findings emphasize that introducing GD screening is not solely a clinical task, but a systems-level intervention requiring coordination across community, facility, and policy domains. Clear referral pathways, structured supervision for frontline staff, and culturally grounded education are critical to translating screening recommendations into practice. These insights may inform clinicians, program managers, and policymakers seeking to introduce or strengthen GD screening in similarly resource-constrained and culturally diverse settings.

### 4.4. Limitations

This study has several limitations. First, as an implementation protocol using de-identified aggregated program data, the findings are context-specific and may not be generalizable beyond rural indigenous communities in Guatemala. All quantitative results reflect program-level counts rather than a defined research cohort, and no inferential analyses were performed. Measurement error may have occurred due to the use of POC glucometers which, while reporting plasma-equivalent values, can differ from laboratory venous plasma measurements. Selection bias is possible, as participation in screening was voluntary and may not reflect the broader population. Loss to follow-up limited the completeness of longitudinal outcome data, and the absence of a comparator group prevents direct evaluation of the protocol’s effectiveness relative to standard care. Data collection relied on locally available resources and staff capacity, which may have introduced variability in screening, counseling, and follow-up. Despite these limitations, the iterative implementation process allows for ongoing refinement of the protocol, enhancing its feasibility and sustainability within the target community.

## 5. Conclusions

This implementation process report documents the development and early introduction of a community-centered GD screening protocol in a low-resource, rural Indigenous setting. The findings highlight the importance of engaging local stakeholders, identifying system-level and sociocultural barriers, and applying iterative adaptations to ensure feasibility and acceptability within the local context. Rather than evaluating clinical effectiveness, this work demonstrates how structured implementation approaches can support the introduction of GD screening in settings where standardized protocols and referral pathways are limited or absent. Based on these implementation experiences, we recommend that community-centered GD screening initiatives be developed collaboratively with local stakeholders, tailored to existing health system capacity and community norms, and refined through ongoing feedback. Such an approach may enhance program acceptability, scalability, and sustainability while supporting integration into routine prenatal care. By systematically documenting real-world implementation processes, this report contributes to transferable insights that may inform similar maternal health initiatives in other low-resource settings.

## Figures and Tables

**Table 1:** Stakeholders involved in gestational diabetes screening during the pre-implementation (exploration) phase.

Health Center Location	(1) Santiago Atitlan	(2) Santiago Atitlan	(3) San Juan La Laguna	(4) San Juan La Laguna	(5) San Juan La Laguna	(6) San Pablo La Laguna
Population (habitants)	46,561	46,561	13,973	13,973	13,973	8124
Facility type	MOH clinic	Private NGO	MOH clinic	NGO	NGO	MOH clinic
Level of healthcare	Secondary	Secondary	Secondary	Secondary	Secondary	Secondary
Screening modality	POC fingerstick	POC fingerstick	POC fingerstick	POC fingerstick	POC fingerstick	POC fingerstick
Diagnostic cutoff (mg/dL)	100	92	100	100	90	100
GA cutoff for diagnosis	Any	≥24 weeks	Any	≥24 weeks	>20 weeks	Any
Management	NHS referral	Unclear	NHS referral	NHS referral	NHS referral	NHS referral

MOH = Ministry of Health; NGO = non-governmental organization; GA = gestational age; POC = point-of-care; NHS = National Hospital of Sololá.

**Table 2:** De-identified program-aggregated GD screening data.

Total Number of Women Screened *			103
Women who completed ≥ 2 screenings **			25
Women who completed 1 screening ***			78
Women screened at <24 weeks GA and declined repeat screening at ≥24 weeks GA †			13
Total number of women diagnosed with GD			12
Women who underwent OGTT			9
Women who declined OGTT			6
**Characteristics of Women Screened**			
Age range: 15–46 years old (mean 26.9)			
BMI range: 19–39 (mean 28.6)			
Risk factor	Presence of risk factor (*n*, %)	*n* total recorded ††	*n* total missing ψ
BMI > 30	35 (47.9%)	73	30
Age > 35	17 (23.3%)	103	0
Has first-degree relative with DM	4 (5.5%)	73	30
History of macrosomic infant	4 (5.5%)	73	30
History of stillborn	0 (0%)	73	30
History of >3 miscarriages	0 (0%)	73	30

GA = gestational age; GD = gestational diabetes; OGTT = oral glucose tolerance test; BMI = body mass index; DM = diabetes mellitus. * From March 2023–August 2024. ** One screening at <24 weeks GA and one at ≥24 weeks. GA (per protocol). *** Completed one screening at either <24 weeks GA or ≥24 weeks GA (but not both). † Number of women completing screenings at <24 weeks GA and whom at ≥24 weeks GA, were identified, contacted, and offered screening but they explicitly declined. †† Number of women for whom risk factor was documented. ψ Number of women for whom risk factor was not documented.

## Data Availability

No datasets were generated or analyzed during the current study. The findings presented were derived from the implementation process and contextual observations, not from database analysis.
